# Advanced Nanoprobe Strategies for Imaging Macrophage Polarization in Cancer Immunology

**DOI:** 10.34133/research.0622

**Published:** 2025-02-21

**Authors:** Junjie Zhang, Kaiyuan Tang, Yongbin Yang, Dongliang Yang, Wenpei Fan

**Affiliations:** ^1^ School of Fundamental Sciences, Bengbu Medical University, Bengbu 233030, P.R. China.; ^2^ State Key Laboratory of Organic Electronics and Information Displays & Institute of Advanced Materials (IAM), Nanjing University of Posts & Telecommunications, Nanjing 210023, P.R. China.; ^3^ Interdisciplinary Eye Research Institute (EYE-X Institute), Bengbu Medical University, Bengbu 233030, P.R. China.; ^4^ State Key Laboratory of Flexible Electronics (LoFE) & Institute of Advanced Materials (IAM), School of Physical and Mathematical Sciences, Nanjing Tech University (NanjingTech), Nanjing 211816, P.R. China.; ^5^ State Key Laboratory of Natural Medicines and Jiangsu Key Laboratory of Drug Discovery for Metabolic Diseases, Center of Advanced Pharmaceuticals and Biomaterials, China Pharmaceutical University, Nanjing 211198, P.R. China.

## Abstract

Macrophages are ubiquitous within the human body and serve pivotal roles in immune surveillance, inflammation, and tissue homeostasis. Phenotypic plasticity is a hallmark of macrophages, allowing their polarization into distinct phenotypes M1 (pro-inflammatory, anti-tumor) and M2 (anti-inflammatory, pro-tumor) in response to local microenvironmental cues. In tumor tissues, the polarization of tumor-associated macrophages profoundly shapes the tumor microenvironment, influencing tumor progression, immune evasion, and metastasis. Therefore, the ability to image and monitor macrophage polarization is essential for comprehending tumor biology and optimizing therapeutic strategies. With the rapid advancement of nanomedicine, a diverse array of nanoprobes has been engineered to specifically target tumor-associated macrophages, offering new avenues for noninvasive in vivo imaging and real-time monitoring of macrophage dynamics within the tumor microenvironment. This perspective highlights recent advancements in macrophage-targeting nanoprobes for imaging macrophage polarization both in vitro and in vivo. It also addresses the current challenges in the field, such as enhancing probe sensitivity, specificity, and biocompatibility, while outlining the future directions for the development of next-generation nanoprobes aimed at precision oncology.

Immunotherapy has emerged as one of the most promising approaches in cancer treatment, leveraging the body’s own immune system to combat malignancies and offer long-lasting protection against recurrence [1–3]. Central to this process is the infiltration and activation of immune cells within the tumor microenvironment (TME), which are critical determinants of tumor progression and therapeutic outcomes [4–6]. Among the various immune cells, macrophages stand out as key players in shaping the TME, and their phenotypic plasticity, manifested as polarization into M1 (anti-tumor) and M2 (pro-tumor) states, has profound implications for both tumor behavior and patient prognosis [7–11]. As such, understanding the dynamic shift between M1 and M2 macrophages in situ is crucial for the development of more effective immunotherapeutic strategies.

Traditional diagnostic methods, such as histological analysis and flow cytometry, are limited in their ability to provide real-time, noninvasive insights into the polarization state of macrophages within tumors. These methods carry inherent risks, including tissue damage, bleeding, and the potential dissemination of cancerous cells [12]. Consequently, the demand for more refined and minimally invasive imaging tools is greater than ever. In this regard, the development of nanoscale imaging probes has revolutionized our ability to monitor the TME, offering a promising approach for tracking macrophage polarization in vivo [13–15]. Nanoprobes are particularly attractive because of their ability to exploit the enhanced permeability and retention effect, allowing for targeted delivery to tumor sites. Upon reaching the tumor, these probes can be internalized by macrophages, enabling specific monitoring of their polarization status [16–18]. The unique physicochemical properties of nanoprobes, such as their tunable size, surface characteristics, and optical properties, further enhance their performance, offering high sensitivity, low detection limits, and robust resistance to background interference [19,20]. These attributes make them highly suitable for in vivo imaging applications, with real-time monitoring of macrophage behavior poised to provide critical insights into tumor progression and treatment response [21,22].

Among the various types of nanoprobes, fluorescent and chemiluminescent probes have been at the forefront of research in the field. Fluorescent probes are known for their high sensitivity and spatial resolution, capable of detecting events down to the single-molecule level [23,24]. However, challenges such as tissue autofluorescence and the low signal-to-background ratio (SBR) often hinder their performance in complex biological systems [25,26]. In contrast, chemiluminescence (CL) imaging, which does not require optical excitation, provides a promising solution by obviously reducing background noise. This approach allows for a higher SBR, improving imaging clarity and sensitivity [27–29]. Huang et al. [30] developed a series of near-infrared (NIR) chemiluminescent molecules (DPDO, DPDS, BPDO, and BPDS) based on an adamantyl-1,2-dioxetane framework. Among these, DPDO demonstrated exceptional CL properties, including the highest quantum yield (2.7% einstein/mol) and the longest half-life (approximately 7.7 h). Building on these impressive characteristics, the authors engineered DPDO into a tandem-locked NIR chemiluminescent probe (DPDGN), which is activated in the presence of both nitric oxide (NO) and γ-glutamyltransferase. This innovative design resulted in a remarkable 26.6-fold enhancement of the 725-nm CL signal, providing a powerful tool for tracking macrophage polarization in the TME. Flow cytometry analysis and tumor-bearing mouse models revealed that DPDGN’s CL signal was closely correlated with the degree of tumor-associated macrophage polarization toward the M1 phenotype, highlighting its potential as a noninvasive, real-time biomarker for monitoring anti-tumor immune responses. Moreover, DPDGN can undergo further surface modification to attain enhanced targeting capabilities, better biosafety, an improved signal-to-noise ratio, and other desirable characteristics. Additionally, delving deeper into the potential work mechanism of DPDGN is crucial for assessing its practical application potential.

While CL has shown great promise for imaging M1 macrophages, the challenge of monitoring the M2 macrophage population, which is often associated with tumor progression and immune suppression, remains an area of intense research. The M2 phenotype is particularly difficult to target due to its subtle and dynamic nature within the TME. However, recent innovations have begun to address this challenge. Yuan et al. introduced a novel NO-responsive nanoprobe (NRP@M-PHCQ) designed to specifically target M2-like macrophages (Figure A). This system incorporates NO-responsive near-infrared II probes (NRPs) encapsulated in polymeric micelles formed from amphiphilic block copolymers that contain mannose and hydroxychloroquine (HCQ) [31]. The mannose groups endow targeted delivery to M2 macrophages, while HCQ polarizes M2 macrophages toward M1 phenotype macrophages by enhancing NO secretion. The probe’s activation, triggered by NO secretion, leads to the emission of NIR light in the 900- to 1,200-nm range, allowing for sensitive imaging of macrophage dynamics. In vivo experiments demonstrated that NRP@M-PHCQ could sensitively track early tumor metastases to lymph nodes and lungs, outperforming traditional bioluminescence-based imaging techniques. Furthermore, the polarization of M2 macrophages into M1 phenotype macrophages suppressed tumor cell growth and almost completely eradicated metastatic lung tumors. This work offers a novel strategy for sensitively monitoring and inhibiting early tumor metastasis through targeting NO-responsive probes and visually demonstrates the critical role of M2-like macrophages in early tumor metastasis. Compared to traditional in vitro subtype detection methods, NRP@M-PHCQ probes hold greater application prospects for the long-term dynamic monitoring of primary tumors and tumor metastasis.

**Figure.**
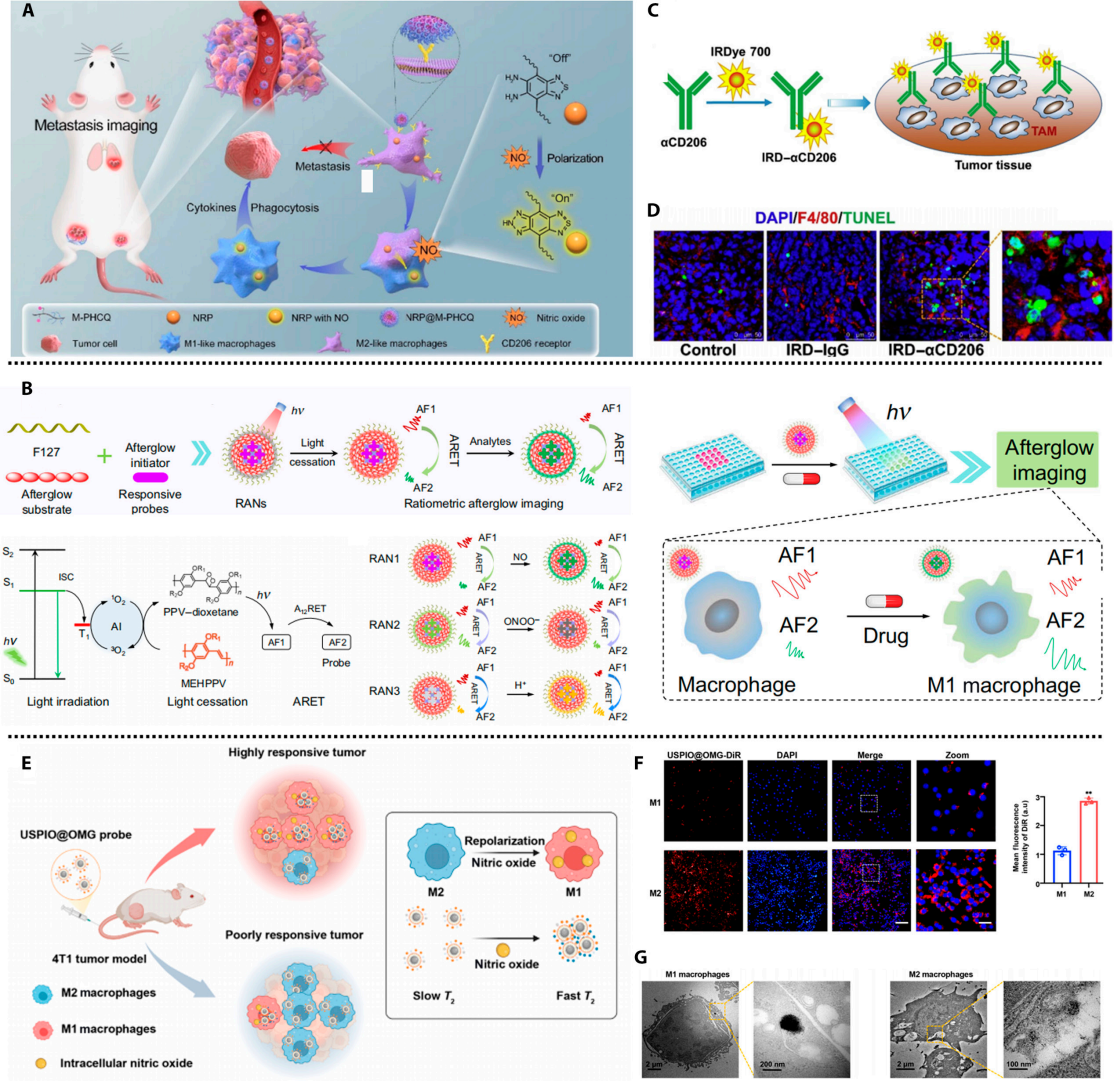
(A) Illustration of the fabrication and mechanism of action of the NRP@M-PHCQ nanoplatform. Reproduced from Ref. [31] with permission from the American Chemical Society, copyright 2023. NRP@M-PHCQ, NO-responsive near-infrared II probe encapsulated in polymeric micelles formed from amphiphilic block copolymers that contain mannose and hydroxychloroquine. (B) Schematic representation of the afterglow resonance energy transfer (ARET)-based ratio nanoplatform. ISC, intersystem crossing; PPV, poly(phenylene vinylene); MEHPPV, poly[2-methoxy-5-(2-ethylhexyloxy)-1,4-phenylenevinylene]; AF1, afterglow luminescence from MEHPPV; AF2, afterglow luminescence from NO-responsive nanoprobe. Reproduced from Ref. [37] with permission from Springer Nature, copyright 2022. (C) Synthetic diagram of IRDye700-conjugated antibody (IRD–αCD206) for tumor-associated macrophage (TAM) targeting. (D) Immunofluorescence images showing apoptotic cells and macrophages treated with different groups. Reproduced from Ref. [40] with permission from Elsevier, copyright 2016. (E) Mechanistic illustration of the USPIO@OMG nanoprobe. (F) Immunofluorescence images of M1 and M2 macrophages incubated with USPIO@OMG nanoparticles. (G) Depiction of the cellular distribution and aggregation of USPIO@OMG nanoparticles in M1 and M2 macrophages. Reproduced from Ref. [42] with permission from the American Chemical Society, copyright 2023. USPIO@OMG, NO-responsive magnetic resonance imaging nanoprobe based on ultrasmall paramagnetic iron oxide nanoparticles modified with a combination of *o*-phenylenediamine, mannose, and gadolinium chelates; DAPI, 4′,6-diamidino-2-phenylindole; TUNEL, terminal deoxynucleotidyl transferase dUTP nick end labeling; IgG, immunoglobulin G; RAN, NO-responsive afterglow nanoprobe; DiR, 1,1-dioctadecyl-3,3,3,3-tetramethylindotricarbocyaineiodide.

Afterglow luminescence is an intrinsic emission pathway where molecules release stored energy after optical stimulation, eliminating the need for continuous excitation. This technique holds great promise for background-free molecular imaging in vivo, offering enhanced sensitivity and clarity in the complex, high-background environment of the TME [32,33]. However, challenges remain, particularly in the time-dependent decay characteristics of afterglow materials, which complicate precise quantification. Additionally, the limited reactivity and structural inertness of these materials present technical hurdles in optimizing their functionality for in vivo applications [34–36]. Liu et al. made notable strides in overcoming these limitations by developing a novel afterglow resonance energy transfer strategy. The result is an innovative NO-responsive afterglow nanoprobe (RAN1), designed to address the challenge of detecting macrophage polarization in the TME (Figure B) [37]. Upon activation by NO, a molecule produced by activated M1 macrophages, RAN1 exhibits a distinct afterglow shift—decreasing at 600 nm and increasing at 830 nm. This specific response allows for high signal-to-noise ratio imaging, enabling the targeted detected NO released from M1 phenotype macrophages. By leveraging this strategy, the researchers created a universal ratiometric afterglow nanoprobe platform (RAN), capable of detecting a range of analytes, including NO, peroxynitrite (ONOO^−^), and pH. These afterglow probes not only address the attenuation of afterglow intensity but also eliminate the interference from external factors such as laser power, irradiation time, and exposure time, obviously enhancing the reliability of in vivo imaging and achieving an approximately 1,200-fold increase in the SBR. Consequently, the ratiometric afterglow nanoprobe platform can accurately reflect the changes in biomarkers of macrophage polarization, enabling real-time dynamic assessment of the extent of tumor immunotherapy, which provides a reliable parameter for predicting the outcomes of immunotherapeutic interventions. However, the study validated the feasibility of the NO-responsive probe RAN1, while the reliability of the pH-and ONOO^−^-responsive probes has not been experimentally demonstrated. Furthermore, it remains to be experimentally explored whether designing afterglow imaging probes with multisource responsiveness would better locate macrophages in different polarization states and improve the accuracy of imaging. Although afterglow imaging exhibits broad future application potential, its existing limitations still require the development of more feasible solutions.

Additionally, another innovative approach for monitoring macrophage polarization is the targeting of macrophage-specific markers such as CD206, the macrophage mannose receptor, which is predominantly expressed on M2 macrophages [38,39]. Recent research by Zhang et al. [40] demonstrated the power of targeting this receptor using an NIR phthalocyanine dye conjugated to a monoclonal anti-CD206 antibody, creating the IRDye700-conjugated CD206 antibody (IRD–αCD206) probe (Figure C). This probe has proven highly effective in noninvasively tracking tumor-associated macrophage infiltration in tumors using NIR fluorescence imaging. Their studies in 4T1 tumor-bearing mice revealed that sorafenib treatment led to M2 macrophage polarization, which was successfully visualized via the IRD–αCD206 probe (Figure D). Importantly, the researchers also demonstrated the potential therapeutic utility of the IRD–αCD206 probe by incorporating photodynamic therapy. When irradiated, the probe’s photodynamic properties inhibited tumor growth and prevented metastatic spread to the lungs, highlighting a promising strategy for simultaneously imaging and treating tumors. However, phthalocyanine dyes may experience photobleaching or photodegradation under conditions of high light intensity or prolonged excitation or within complex pathological environments. This will significantly diminish the imaging performance. Therefore, the design of highly stable fluorophore-based probes is significant for long-term real-time detection. Moreover, the targeting ability of the probe and the integration of diagnosis and treatment are of great importance for the progress of personalized treatment in the later stage.

While fluorescence imaging remains a cornerstone of molecular imaging due to its high sensitivity and spatial resolution, it suffers from significant limitations in the context of deep tissue imaging. Fluorescence probes are highly susceptible to tissue autofluorescence, which diminishes the SBR and restricts their use in complex biological systems, such as the TME. Despite the clinical utility of imaging techniques like positron emission tomography (PET), single-photon emission computed tomography (SPECT), and computed tomography, these modalities continue to face challenges related to spatial resolution, sensitivity, and their inability to provide cell-specific information [41]. Magnetic resonance imaging, however, stands out due to its exceptional soft tissue contrast and anatomical details, offering great potential for the development of responsive contrast agents to assess molecular targets in vivo [41]. Liu et al. [42] developed a NO-responsive magnetic resonance imaging nanoprobe (USPIO@OMG) based on ultrasmall paramagnetic iron oxide nanoparticles (USPIO) modified with a combination of *o*-phenylenediamine, mannose, and gadolinium chelates (Figure E). This probe’s ability to react with NO molecules results in charge reversal and the formation of aggregated structures that specifically target M2 macrophages (Figure F and G), thereby enabling differentiation between macrophage subtypes in the TME. Flow cytometry analysis revealed that the mean fluorescence intensity of M2 macrophages targeted by the probe was 2.53 times higher than that of M1 macrophages (Figure F), demonstrating a robust, concentration-dependent response. The T1 and T2 relaxation changes induced by the USPIO@OMG nanoprobe can be used to detect the phenotypic changes of macrophages. This study further evaluates the tumor response of USPIO@OMG to Toll-like receptor agonist R848-mediated immunotherapy and x-ray-mediated radiotherapy, allowing macrophage polarization detection techniques to provide immune-related guidance and insights for existing treatments.

Currently, significant advances have been made in the detection of macrophage types within tumors through the use of different imaging techniques. Although these technologies demonstrate great potential in clinical applications, the field still confronts some substantial challenges. Firstly, different imaging strategies possess distinct advantages and disadvantages (Table). Consequently, the development of multimodal imaging, high-sensitivity, and integrated diagnostic and therapeutic probes is crucial. These probes can better align with various detection instruments and enable synchronous diagnosis and treatment [43,44]. Secondly, improving the signal-to-noise ratio, enhancing the target-to-background contrast, and achieving precise identification of macrophage subtypes are crucial factors that can obviously enhance the accuracy and reliability of macrophage imaging. Thirdly, optimizing the biocompatibility and minimizing the toxicity of these nanoprobes are indispensable steps for their safe clinical application. Although some studies have demonstrated the short-term biosafety of nanoprobes, long-term biotoxicity testing is still lacking and requires urgent improvement. Fourthly, streamlining the synthesis process of nanoprobes and establishing probe testing standards are vital for meeting the requirements of industrial-scale production. Simplifying the synthesis process not only cuts costs but also enhances product consistency and reproducibility while avoiding potential harm to the natural environment. In cancer treatment, macrophage polarization detection technology can optimize immune cell therapy, target the immune environment of cancer cells, formulate specific immunotherapy strategies, enhance the selectivity of cancer cell treatment, minimize damage to normal cells, and provide a means to monitor the progress of cancer immunotherapy. Moreover, as various nanoprobes enter clinical trials, integrating the nanoprobe platform with tumor treatment strategies will boost the efficiency of disease treatment and expedite its clinical translation.

**Table.** The advantages and disadvantages of different imaging strategies for nanoprobe-based detection of macrophage polarization

Imaging modality	Advantages	Disadvantages
Fluorescence imaging	Fast, real-time, sensitive, noninvasive, without ionizing radiation, capable of simultaneously monitoring distinct fluorescence signals	Restricted penetration depth, phototoxicity, photobleaching, background fluorescence signals
Chemiluminescence imaging	High sensitivity, strong signal intensity, no background interference, noninvasive, lower phototoxicity, suitable for deep tissue imaging, high temporal resolution	Limited light sources, signal attenuation, complex probe design, overly concentration-dependent signals
NIR imaging	NIR-II provides good tissue penetration, lower phototoxicity, high sensitivity, high signal-to-noise ratio, noninvasive	Penetration depth of NIR-I, background interference
Afterglow imaging	Long-lasting imaging, tunable emission from the visible to NIR range, repeatable activation, long afterglow lifetime, no in situ excitation required, no tissue background signal interference, high signal-to-noise ratio	Inevitable signal decay after light source cessation, structural inertness of afterglow materials complicating probe design, lack of suitable probe design strategies, fluorescent signals susceptible to multiple factors
Computed tomography	Noninvasive, good penetration, high clinical applicability	Radiation exposure, low resolution, poor sensitivity, and potential toxicity of probes
Magnetic resonance imaging	Extremely high resolution, noninvasive, no ionizing radiation	Bulky and expensive equipment, poor spatial resolution
PET/SPECT	Rapid quantification of radionuclide-labeled nanomaterial distribution in organs and tissues, high sensitivity	Radioactivity and inability to provide precise anatomical localization

NIR, near-infrared; PET, positron emission tomography; SPECT, single-photon emission computed tomography

## Data Availability

All data needed to evaluate the conclusions of the study are available in the manuscript.
